# Beneficial effects of omega-3 fatty acids in the proteome of high-density lipoprotein proteome

**DOI:** 10.1186/1476-511X-11-116

**Published:** 2012-09-16

**Authors:** Elena Burillo, Rocío Mateo-Gallego, Ana Cenarro, Sarah Fiddyment, Ana M Bea, Inmaculada Jorge, Jesús Vázquez, Fernando Civeira

**Affiliations:** 1 Laboratorio de Investigación Molecular and Unidad de Lípidos, Hospital Universitario Miguel Servet, Instituto de Investigación Sanitaria (IIS), Zaragoza, Spain; 2 Laboratorio de Proteómica Cardiovascular, Centro Nacional de Investigaciones Cardiovasculares (CNIC), Madrid, Spain; 3 Red Cardiovascular RECAVA, Fondo de Investigación Sanitaria, Instituto de Salud Carlos III, Madrid, Spain

**Keywords:** Omega-3 poly-unsaturated fatty acids, HDL proteome, Cardiovascular risk

## Abstract

**Background:**

Omega-3 poly-unsaturated fatty acids (ω-3 PUFAs) have demonstrated to be beneficial in the prevention of cardiovascular disease, however, the mechanisms by which they perform their cardiovascular protection have not been clarified. Intriguingly, some of these protective effects have also been linked to HDL. The hypothesis of this study was that ω-3 PUFAs could modify the protein cargo of HDL particle in a triglyceride non-dependent mode. The objective of the study was to compare the proteome of HDL before and after ω-3 PUFAs supplemented diet.

**Methods:**

A comparative proteomic analysis in 6 smoker subjects HDL before and after a 5 weeks ω-3 PUFAs enriched diet has been performed.

**Results:**

Among the altered proteins, clusterin, paraoxonase, and apoAI were found to increase, while fibronectin, α-1-antitrypsin, complement C1r subcomponent and complement factor H decreased after diet supplementation with ω-3 PUFAs. Immunodetection assays confirmed these results. The up-regulated proteins are related to anti-oxidant, anti-inflammatory and anti-atherosclerotic properties of HDL, while the down-regulated proteins are related to regulation of complement activation and acute phase response.

**Conclusions:**

Despite the low number of subjects included in the study, our findings demonstrate that ω-3 PUFAs supplementation modifies lipoprotein containing apoAI (LpAI) proteome and suggest that these protein changes improve the functionality of the particle.

## Background

In the last decades, different observational and intervention studies have demonstrated a beneficial effect of diets enriched in omega-3 poly-unsaturated fatty acids (ω-3 PUFAs) in the prevention of cardiovascular disease (CVD). Most common ω-3 PUFAs are α-linolenic acid C18:3 n-3, present in plants, and eicosapentaenoic acid (EPA) and docosahexaenoic acid (DHA), found in fish oil. The dose–response (3–4 g/day) hypotriglyceridemic effect (fasting and postprandial) is the best defined metabolic effect of ω-3 PUFAs [1]. The mechanism for this lipid lowering effect seems to be due to activation of peroxisome proliferator-activated receptors (PPAR) [2].

Other potential beneficial effects of ω-3 PUFAs include reduction of susceptibility to ventricular arrhythmia [3]; antithrombogenic and antioxidant effect [4]; retardation of the atherosclerotic plaque growth by reduced expression of adhesion molecules and platelet-derived growth factor [4] and anti-inflammatory effect [5]; promotion of endothelial relaxation by induction of nitric-oxide production, and mild hypotensive effect [6]. All these effect are explained because diet EPA and DHA are rapidly incorporated into the cellular membrane phospholipids where carry out their potential attributed actions [7].

Interestingly, several of the beneficial effects attributable to ω-3 PUFAs have also been linked to HDL. HDL has been involved in anti-thrombosis and endothelial dysfunction [8], anti-inflammatory effect [9], inhibition of lipoprotein oxidation [10,11], regulation of the complement system, inhibition of proteolysis and regulation of acute phase response [12]. Omega-3 PUFAs do not substantially modify the cholesterol transported by the HDL but, it is now assumed that HDL cholesterol is not a good marker of the functional capacities of the particle. Recent studies have confirmed that protein content of HDL is complex, and it is more related with certain anti-atherogenic properties of HDL than HDL cholesterol [13].

The hypothesis of this study was that ω-3 PUFAs could modify the protein cargo of HDL particle in a triglyceride non-dependent mode. Regarding this, the effect of ω-3 PUFAs would be directly related, at least in part, to cardioprotective actions of HDL particle. In order to elucidate this hypothesis, a comparative proteomic analysis of HDL particle before and after a ω-3 PUFAs enriched diet has been performed in a smoking healthy male volunteers group, a population characterised by dysfunctional HDL particles [14] in which high fish consumption reduces the CVD risk associated with smoking [15].

## Results and dicussion

### Results

#### Clinical, biochemical and dietary characteristics

The study group was composed of 6 male smokers, all of whom completed the experiment. Tobacco consumption was used as a model to study dysfunctional HDL particle. Their mean age was 43.0 (28.5-51.0) (median (interquartile range)) and their mean tobacco consumption was 25.5 (20.0-31.3) cigarettes per day. Table 1 shows the main clinical variables at baseline and at the end of the intervention. As expected, due to the low dose of ω-3 PUFAs supplementation, there were not changes in the lipids parameters. Baseline diet assessment showed a mean energy intake of 2847 (2671–2991) kcal with a high consumption of total fat 37.6 (35.8-40.4)%, a low intake of carbohydrates 42.8 (39.0-47.6)%, and a protein intake of 14.8 (14.3-15.8)%. Marine and non-marine omega-3 fatty acids intake was 0.60 (0.25-1.07) g/day and 1.36 (1.19-1.98) g/day, respectively. Physical activity questionnaire results showed a mean of 64.2 (36.5-81.3) METs/week. Dietary parameters and exercise level were stable throughout the study as FFQ and physical activity questionnaire performed at the end of the study revealed (data not shown).

**Table 1:** Clinical characteristics of study subjects at base line and after 5 weeks of ω-3 PUFAs supplementation

	**Basal situation**	**Post w-3 PUFAs supplementation**	***p*Value**
**Body mass index, Kg/m**^**2**^	26.1 (24.0-28.0)	26.3 (24.1-27.9)	0.753
**Waist circunference, cm**	95.0 (87.1-98.5)	95.0 (87.0-101)	0.785
**Systolic blood pressure, mmHg**	134 (115–139)	122 (107–129)	0.042
**Dyastolic blood pressure, mmHg**	78.0 (67.7-85.5)	77.5 (67.7-81.7)	0.109
**Total cholesterol, mg/dL**	208 (186–232)	214 (190–249)	0.345
**LDL cholesterol, mg/dL**	136 (104–170)	144 (107–184)	0.173
**HDL cholesterol, mg/dL**	49.0 (41.0-61.5)	49.0 (45.7-59.2)	0.496
**Non-HDL cholesterol, mg/dL**	155 (122–191)	162 (122–205)	0.528
**Triglycerides, mg/dL**	92.5 (74.2-127)	92.0 (77.2-111)	0.500
**Apo A-I, mg/dL**	143 (136–166)	150 (146–162)	0.893
**Apo B, mg/dL**	111 (90.6-145)	109 (86.8-139)	0.116
**Lp(a), mg/dL**	57.3 (16.3-59.1)	35.5 (16.3-66.7)	0.273
**CRP, mg/L**	2.55 (1.47-4.45)	3.45 (0.80-5.20)	0.345
**Creatinine, mg/dL**	0.86 (0.82-0.99)	0.94 (0.74-1.04)	0.686
**Glucose, mg/dL**	87.5 (79.0-93.5)	78.5 (71.2-92.0)	0.043

Variables are expressed as median (interquartile range). Apo denotes apolipoprotein. CRP denotes C Reactive Protein.

#### Omega-3 PUFAs compliance

Omega-3 PUFAs supplements were well tolerated by all subjects who did not show any side effect. According to participant’s reports and recounts of empty packages, compliance with the supplements was 100%.

#### Proteomic results

**Figure 1:**
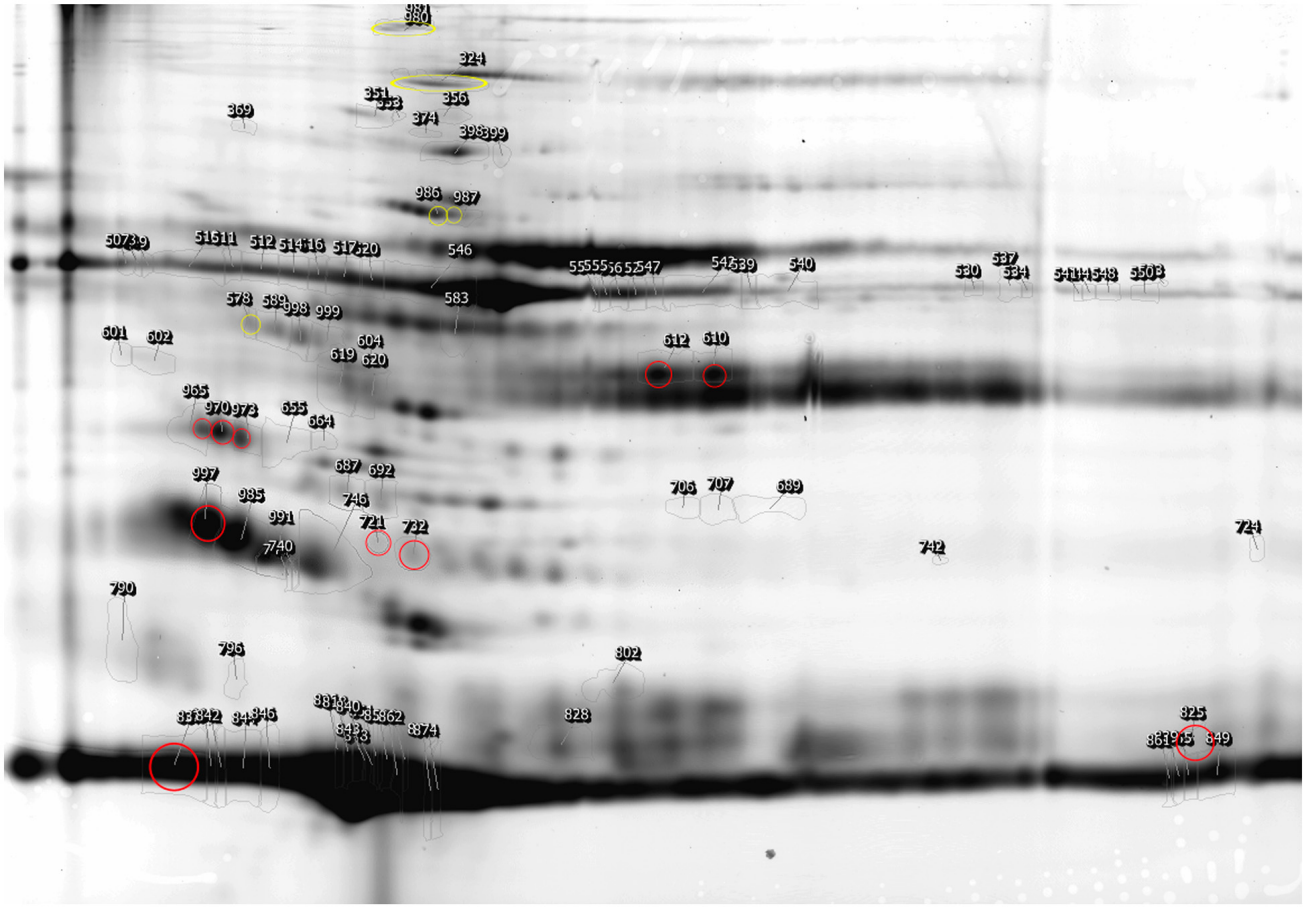
** Differentially expressed protein spots identified by DIGE analysis.** Proteins were extracted as described and separated in pH 3–10 IPG strips for the first dimension and 10% polyacrylamide for the second dimension. The image was acquired on a Typhoon 9400 scanner at 633/670-nm excitation/emission wavelengths. Spots detected by the analysis software are indicated. Ten protein spot-features were found to be significantly up-regulated (red) after the ω-3 PUFAs supplementation and six were significantly down-regulated (blue) in Lp-AI of smoker participants.

**Figure 2:**
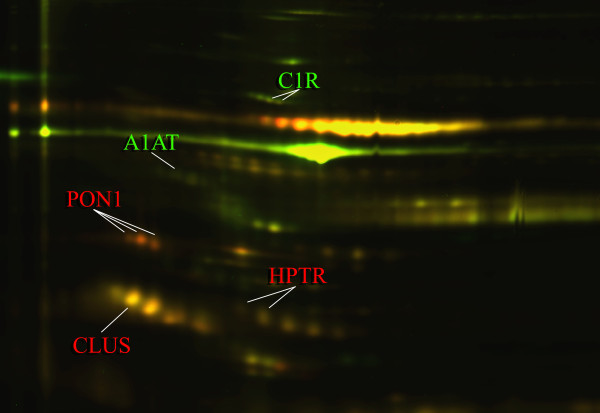
** Representative section of the 2D-DIGE proteome map of basal and after the ω-3 PUFAs supplementation.** Proteins were labeled with Cy3 (basal situation) and Cy5 (after the ω-3 PUFAs supplementation). An internal standard comprised of equal amount of proteins from all samples (basal and after the treatment) was labeled with Cy2 and included in all gels. The green spots indicate downregulated proteins, while the red spots indicate upregulated proteins after the ω-3 PUFAs supplementation. Some of the most representative identified proteins that showed significantly altered expression after ω-3 PUFAs supplementation are indicated with arrows and labeled with the respectives protein entry name.

Proteomic analysis revealed that 28 spots were differentially expressed (p < 0,05) between both study periods. By mass spectrometry, 16 out of the 28 spots were identified: 10 spots were identified up-regulated after ω-3 PUFAs treatment and 6 spots were down-regulated. The up-regulated spots corresponded to apoAI, clusterin, paraoxonase (PON1), fibrinogen β, haptoglobin-related protein (HPTR) and immunoglobulin kappa chain C region; and the down-regulated spots corresponded to fibronectin, α-1-antitrypsin (A1AT), serum albumin, immunoglobulin mu chain C region, complement C1r subcomponent and complement factor H. Mass spectrometry identification results are presented in Table 2 and a representative 2D image is shown in Figure 1. Moreover, in Figure 2 a representative section of the gel labelled with the DIGE dyes is presented. In Additional file 1: Table S1 the normalized volume of each spot in every gel is detailed. To better understand the differences existing between basal and after-treatment HDL proteome, proteins identified were classified by biological function (Table 3).

**Table 2:** Identification results of proteins differentially expressed in basal and after ω-3 PUFAs supplementation

**Spot**	**Protein name**	**ANOVA (p < 0.05)**	**Fold change**	**Protein expression level after ω-3 supplementation**	**UniProt ID**	**Entry name**	**Mascot score**	**Sequence coverage (%)**	**Number of Peptidic masses identified**	**Number of MS/MS identified**	**MW (KDa)**	**pI**
837	Apolipoprotein A-I	0.044	1.18	Up-regulated	P02647	APOA1	87	42	10	1	30.8	5.56
997	Clusterin	0.029	1.3	Up-regulated	P10909	CLUS	735	47	30	8	53	5.89
610	Fibrinogen β	0.048	1.1	Up-regulated	P02675	FIBB	233	49	29	4	56.6	8.54
612	Fibrinogen β	0.045	1.1	Up-regulated	P02675	FIBB	140	45	20	1	56.6	8.54
721*	Clusterin	0.02	1.4	Up-regulated	P10909	CLUS	519	23		11	53	5.89
	Apolipoprotein A-I	0.02	1.4	Up-regulated	P02647	APOA1	478	39		13	30.8	5.56
	Haptoglobin-related protein	0.02	1.4	Up-regulated	P00739	HPTR	477	26		12	39.5	6.63
732	Haptoglobin-related protein	0.015	1.37	Up-regulated	P00739	HPTR	79	31	14	4	39.5	6.63
965	Serum paraoxonase	0.003	1.6	Up-regulated	P27169	PON1	142	15	9	4	39.8	5.08
970	Serum paraoxonase	0.003	1.97	Up-regulated	P27169	PON1	411	56	24	8	39.8	5.08
973	Serum paraoxonase	0.003	1.86	Up-regulated	P27169	PON1	235	31	14	4	39.8	5.08
825*	Ig kappa chain C region	0.036	1.27	Up-regulated	P01834	IGKC	872	86		8	11.6	5.87
	Apolipoprotein A-I	0.036	1.27	Up-regulated	P02647	APOA1	397	52		15	30.8	5.56
578*	α-1-antitrypsin	0.001	0.74	Donw-regulated	P01009	A1AT	364	51		18	46.7	5.59
986*	Complement C1r subcomponent	0.018	0.85	Donw-regulated	P00736	C1R	1601	65		45	80.1	6.21
987*	Serum Albumin	0.032	0.85	Donw-regulated	P02768	ALBU	1114	75		46	69.3	6.3
	Ig mu Chain C region	0.032	0.85	Donw-regulated	P01871	IGHM	987	57		25	49.3	6.8
	Complement C1r subcomponent	0.032	0.85	Donw-regulated	P00736	C1R	784	46		28	80.1	6.2
324	Complement Factor H	0.017	0.72	Donw-regulated	P08603	CFAH	72	16	19	1	14.4	6.21
980*	Fibronectin	0.01	0.66	Donw-regulated	P02751	FN1	1434	26		40	262.5	5.71
981*	Fibronectin	0.002	0.67	Donw-regulated	P02751	FN1	1437	30		48	262.5	5.71

Spots label with a * denotes identification by Orbitrap. Positive fold changes denote proteins up-regulated while negative fold changes denote decrease protein expression after the supplementation. MW denotes Molecular Weight. pI denotes Isoelectric point.

**Table 3:** Biological functions of identified proteins

	**Protein**	**Biological Function**
Increased after ω-3 PUFAs supplementation	ApoAI	Lipid transport and metabolism
	Clusterin	Apoptosis, Complement system regulation, innate immunity
	Fibrinogen β	Coagulation, signal transduction
	Haptoglobin-related protein	Proteolysis
	Serum paraoxonase	Toxic metabolites hydrolysis, inhibition of LDL oxidation
	Immunoglobuling kappa chain C region	Complement system regulation, innate immunity
Decreased after ω-3 PUFAs supplementation	Alpha-1-antitrypsin	Acute phase response, coagulation
	Complement C1r subcomponent	Immune system, complement system regulation
	Complement factor H	Immune system, complement system regulation
	Fibronectin	Acute phase response, angiogenesis, cellular adhesion
	Serum albumin	Transport, regulation of colloidal osmotic pressure, platelet activation
	Immunoglobuling mu chain C region	Innate immunity

#### Confirmation of proteomic results by immunodetection methods

Clusterin increase observed by proteomics was confirmed by immunodetection (p < 0.05). The fold change observed in clusterin measured by ELISA was 2.18 (basal situation: 3.81E-7 (3.72E-7-3.90E-7); after the supplementation: 8.29E-7 (5.06E-7-1.17E-6). Comparison of HDL PON1 between the study situations showed an increased in enzyme activity (fold change: 1.22) after ω-3 PUFAs supplementation, but it did not achieve statistical significance (basal situation: 4.47 (3.10-5.30); after the supplementation: 6.68 (4.24-11.5)).

**Figure 3:**
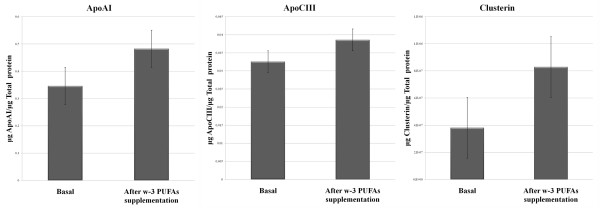
** Expression levels of apoAI, apoCIII and clusterin.** Protein expression levels were divided by total protein concentration in order to normalize values. Expression level of apoAI and apoCIII were measured by MILLIPLEX MAP assays and clusterin levels was measured by ELISA in participants Lp-AI particles. Changes in protein expression level were all of them statistically significant (p < 0,05).

Moreover, Milliplex MAP assays were performed to measure apoAI, apoE and apoCIII, proteins known to be constituents of HDL and to be related to cardiovascular disease. Significant results are shown in Figure 3.

### Discussion

A recent meta-analysis of 29 clinical trials including more than 35,000 high risk cardiovascular patients has explored the effects of ω-3 PUFAs and it has shown a reduction in total mortality associated with the use of these fatty acids [16]. Similar results were obtained from the meta-analysis of 12 studies including 32,779 patients from randomised controlled trials of fish oil as dietary supplements [17]. Both independent studies have concluded that fish oil supplementation was associated with a reduction in deaths from cardiac causes. However, the mechanisms of these pleiotropic effects of fish oil remain mostly unknown [18]. In spite of evident similarities between the protective cardiovascular action of ω-3 PUFAs and HDL, no previous studies had explored the effects of ω-3 PUFAs on protein composition, other than apoAI, on HDL.

Proteomic analysis revealed that some proteins associated to HDL particle were significantly up- or down-regulated in smoking healthy male subjects after 5 weeks consuming a ω-3 PUFAs enriched diet. The down-regulated proteins were mainly related to acute-phase response, complement system regulation and regulation of platelet activation, while proteins up-regulated after the treatment were proteins implicated in lipid metabolism, coagulation, signal transduction, proteolysis regulation, and protection against oxidation.

In this study, we observed an increase in PON1 produced by the ω-3 PUFAs supplementation. PON1 activity was also measured and, although it did not reach statistical significance, activity was higher after the treatment. It is clearly demonstrated that the higher the level of paraoxonase in HDL, higher is the protective effect of the particle [19]. Taken together, this suggests that HDL cardiovascular functions could be improved by PON1 increase. However, it must be further studied.

Interestingly, clusterin, one of the most abundant proteins in HDL, was also up-regulated after ω-3 PUFAs enriched diet. Although the role of clusterin is not yet fully understood, it is known that administration of an oral clusterin-peptide significantly improves HDL anti-inflammatory properties in animal models [20]. The clusterin increase observed in this study supports the cardioprotective effect of ω-3 PUFAs.

Three well-known proteins related to lipid metabolism and transport, apoAI, apoE and apoCIII, were also increased by the ω-3 PUFAs addition. Apolipoprotein AI is the major protein component of HDL. ApoAI protects from atherosclerosis development by its participation in cholesterol efflux and through its antiinflamatory and antioxidant properties. ApoAI by itself is able to reduce oxidized lipids and its inflammatory effects [21-23]. Many lines of evidence indicate that apoE plays a role in modulating atherogenesis promoting cholesterol efflux from macrophages [24]. Studies made in transgenic mice have shown that apoE-deficient animals, which develop hypercholesterolemia and are prone to spontaneous atherosclerosis, show decreased lesion size when overexpressing human apoAI [21], suggesting that apoE and apoAI operate together to optimize mobilization of macrophage cholesterol, a process critical to limiting plaque development. The observation of apoE increasing levels after ω-3 PUFAs in this study is also in agreement with an improvement in the protective properties of the particle. In this study, an increase in apoCIII protein level has been observed after ω-3 PUFAs supplementation. ApoCIII is present in HDL, but it is also a major protein in VLDL and LDL. ApoCIII has been related to elevated VLDL, LDL cholesterol, TG levels and cardiovascular risk [25]. It was demonstrated that the presence of ApoCIII in HDL is related to diminished cholesterol efflux [26]. One possible explanation is that, although no changes in the lipid profile were observed, changes or redistribution of proteins between proatherogenic lipoproteins, VLDL and LDL, to cardioprotective HDL were actually possible.

The complement system is activated by tobacco consumption [27] and in vitro studies indicate that HDL blocks the assembly of the terminal complement attack complex on endothelial cells [28]. Supporting this observation, our data probably indicate that HDL is involved in the regulation of complement activation by tobacco. Decrease in fibronectin and A1AT, two proteins related to acute phase response [12] and induced by smoking [29,30], is also another sign of the improvement in HDL protective capabilities recovered by ω-3 PUFAs supplementation.

As far as we know, this is the first HDL proteomic study carried out with smokers and one of the few proteomic analysis made with ω-3 PUFAs [31-33]. This study suggests that ω-3 PUFAs modify the particle composition in some proteins that have been clearly associated with cardiovascular protection. However, the sample size is small and further analysis is necessary to extract final conclusions. These results uncover a potential mechanism that could, at least partially, explain some of the benefits of ω-3 PUFAs.

## Conclusions

In conclusion, as expected, low doses of omega-3 fatty acid supplementation do not have effect in the lipid profile. However, they modify HDL proteome, suggesting a positive change in the functionality of the particle in smoking men. Functional studies and in-deep proteomic studies will be important for the best knowledge of the particle. If the observed changes in the protein cargo of HDL have clinical implications must be further studied.

## Methods

### Subjects

The study group was composed by 6 unrelated smoking (≥20 cigarettes/day in the last three months) healthy male volunteers who underwent a routine medical examination in the Hospital Universitario Miguel Servet (Zaragoza, Spain). The study was approved by the local ethical committee and informed consents were obtained from all participant. Clinical examination and blood tests were performed. Inclusion criteria were: age 16–65 years old and normolipidemia defined as cholesterol LDL < 190 mg/dL and triglycerides (TG) < 150 mg/dL. Exclusion criteria were: current use of drugs that modify lipid or glucose metabolism; anti-inflammatory drugs, included statins, fibrates, ezetimibe, resins, aspirin, nonsteroidal drugs, corticoids, immunodepressors, and vitamin complexes; current acute illness (including hepatic illness, diabetes mellitus, kidney illness, cancer and thyroid illness non controlled); parental history of dyslipidemia and ω-3 PUFAs allergy; alcohol consumption (> 30 g/day of ethanol) and any condition that, in the researcher opinion, could interfere with the study.

### Study design

This study was designed as a 5 weeks open study in which 2 g/day (1 g/capsule twice a day) of ω-3 PUFAs, in the commercial format of Omacor© (Ferrer Internacional), were administrated to participants. Basal and after the 5 weeks of supplementation, the lipid profile and the HDL proteome were analyzed.

### Plasma samples

Ten mL of blood before and after ω-3 PUFAs enriched diet were collected on Vacutainer tubes with EDTA as anticoagulant from fasted (≥ 12 hours) volunteers. Sodium azide and the protease inhibitor Pefabloc SC (Roche) were added to plasma at a concentration of 1.5 and 0.5 mM, respectively. All samples were stored at −80°C until they were processed.

### Quantification of lipids and lipoproteins

Total serum cholesterol and triglyceride levels were quantified enzymatically with a Beckman Synchron CX7 analyzer (Boehringer Mannheim, Ingelheim am Rhein, Germany). HDL cholesterol was measured after precipitation of apolipoprotein B-containing lipoproteins with Mg^++^ phosphotungstate (Boehringer Mannheim, Ingelheim am Rhein, Germany). LDL cholesterol was calculated by the Friedewald formula [34].

### Dietary and physical activity assessment

Before the study, participants were instructed to maintain their usual dietary and physical activity patterns throughout all study to avoid changes that could alter the HDL composition. At baseline and final visits, a Spanish validated 137-item food frequency questionnaire (FFQ) and The Minnesota Leisure Time Physical Activity Questionnaire were performed [35,36]. The FFQ included the consumption frequency of each of the 137-item food by choosing between nine possibilities (from never or less than once per month to six or more times per day) and the portion size. The total energy and nutrient intake were calculated based on previously validated Spanish food composition tables [35].

### Isolation of HDL by Fast Protein Liquid Chromatography-affinity cromatography (FPLC-AC)

FPLC-AC analysis was carried out on an ÄKTA FPLC system (GE Healthcare, Waukesha, WI, USA) equipped with a fraction collector. Briefly, fractions of 2 mL of plasma from a single sample were applied to the anTi-ApoAI affinity column made with a 5 mL HiTrap^TM^ NHS-activated HP (GE Healthcare) coupled to 5 mg of antibody against human apoAI (BioDesign International). Firstly, 10 mL of PBS were used to ensure the equilibration of the column. The binding reaction was performed in a saline buffer (0,1 M NaHCO_3_, 0,5 M NaCl, 1 mM EDTA, pH 8,0) at a flow rate of 1 mL/min with a maximum pressure of 0,5 MPa. When the non-binding fraction was washed, buffer was changed to elution buffer (0,1 M glycine, 0,5 M NaCl, 10% dioxane, pH 2,8) at the same flow rate. HDL was collected using a Frac 900 fraction collector (GE Healthcare) at 4°C as a single 5 mL fraction over 1 mL of equilibration buffer (0,5 M Tris–HCl, pH 8) to quickly neutralize the acid pH and to prevent protein degradation. HDL fraction was then concentrated to 200 μL using Amicon Ultra 10 KDa filters (Millipore, Billerica, MA, USA) at 3000 rpm for 35–40 minutes at 4°C. Total protein concentration was calculated by RC/DC Protein assay (BioRad). Finally, samples were treated with the 2-D Clean-Up Kit (GE Healthcare).

### Fluorescence labelling

Three groups were defined for labelling: pooled internal standard, before ω-3 PUFAs and after ω-3 PUFAs enriched diet. Labelling was made following the protocol provided in the kit. A total of 50 μg of each type of HDL sample was labelled with one of the three CyDye DIGE Fluors (GE Healthcare). CyDyes were reconstituted in anhydrous dimethylformamide and combined with samples at a ratio of 400 pmol of CyDye to 50 μg of protein. Labelling was performed on ice and in the dark for 30 min. The reaction was then quenched by incubating with 1 μL of 10 mM lysine on ice and in the dark for 10 min.

### 2-D Gel Electrophoresis

Firstly, immobilized pH gradient (IPG) strips were equilibrated in Destreak rehydratation solution (GE Healthcare) in which anfolites were added. The three labelled protein samples were combined and were focused on 24 cm, 3–10 IPG strips (GE Healthcare) using an IPGphor focusing apparatus (GE Healthcare, Waukesha, WI, USA). Once first dimension electrophoresis had finished, IPG strips were equilibrated with dithiothreitol (DTT) and iodoacetamine buffers. Then, proteins were separated by electrophoresis on 10% polyacrylamide Tris-glycine gels using an Ettan DALT II System (GE Healthcare, Waukesha, WI, USA).

### DIGE Analysis

The gels were scanned with a Typhoon 9400 scanner (GE Healthcare, Waukesha, WI, USA). Spot detection, quantification and image matching were performed with Progenesis SameSpots software (Nonlinear Dinamics, Newcastle upon Tyne, UK). Finally, 2-D gels were fixed in 30% methanol, 7.5% acetic acid, and stained with a silver nitrate protocol compatible with mass spectrometry for protein identification. Relative protein quantification of HDL samples before and after ω-3 enriched diet was performed using Progenesis SameSpots software (Nonlinear Dinamics, Newcastle upon Tyne, UK). The Cy2-labelled pooled internal standard on every gel allowed accurate relative quantification of protein spot features across different gels. Student's *t*-test was used to identify differences in relative abundances of protein spot-features.

### In-gel digestion

Protein spots were excised manually from 2-D gels. Briefly, spots were washed with water, ammonium bicarbonate (25 mM NH_4_HCO_3_), acetonitrile (ACN) and a freshly made mix of potassium ferricyanide 30 mM and sodium thiosulfate 100 mM to eliminate silver. Next, samples were reduced and alquilated by incubation with DTT (10 mM) at 60°C during 45 min followed by incubation with iodoacetamide (50 mM) at room temperature during 30 minutes. Finally, proteins were trypsin digested overnight at 37°C (2,5 ng/μl, ratio enzyme:protein 1:20, Trypsin gold, Promega). Digestion was stopped by addition of 0.5% trifluoroacetic acid (TFA) and tryptic peptides were extracted sequentially with increasing concentrations of ACN in H_2_O. Peptides were concentrated and desalted by passing them through ZipTip C18 columns (Millipore) following the manufacturer’s instructions and eluting with 50%ACN/0,1%TFA/H_2_O.

### Mass spectrometry analyses

Sample (0,4 μl) and matrix (0,8 μl saturated solution of alpha-*Cyano*-4-hydroxycinnamic acid in 50% ACN/0.1% TFA/H_2_O) were spotted in duplicate onto a Opti-Tof 384 well insert plate (Applied Biosystems, Carlsbad, CA, USA). MALDI-TOF MS was performed using a 4800plus MALDI-TOFTOF (Applied Biosystems, Carlsbad, CA, USA) in the reflector mode with accelerating voltage of 20 kV, mass range of 800 to 4000 Da, 1000 shots/spectrum and laser intensity of 2832. MSMS spectra were performed automatically on twenty of most intense precursors, with 1000 shots/spectrum and laser intensity of 3700. Spectra were calibrated externally using a standard protein mixture (4700 Calmix, Applied Biosystems).

Alternatively, samples were dried and resuspended in 0.1% formic acid and analysed by LC-MSMS in a nano Acquity (Waters, Milford, MA, USA) coupled to an OrbitrapVelos (ThermoScientific, Waltham, MA, USA). Sample was injected in a C18 phase reverse column (75 μm Øi, 10 cm, nano Acquity, 1.7 μm BEH column, Waters) in a gradient of 40-60% buffer B during 5 minutes at a flow rate of 250 nl/min (A: 0.1% formic acid; B: ACN/0.1% acid formic). Eluted peptides were ionized by ESI (PicoTipTM, New Objective 2000 V). Peptide mases were analized in the Orbitrap in full scan (m/z 350–1700) and the 5 most abundant peptides were selected for collision-induced dissociation fragmentation using helium as collision gas. Data were extracted with software Thermo Xcalibur (v.2.1.0.1140).

### Protein identification

Proteins were identified using the search engine Mascot and the Uniprot database. Search parameters used were: human, missed cleavage 1, fixed modifications carbamidomethyl (cysteines) and peptide tolerance 0.2 Da (MS) 0.3 Da (MSMS). Proteins with a score above 61 were considered a positive hit.

### Human paraoxonase and complement C3 component ELISA assays

Two commercial ELISA assays against paraoxonase (Uscn Life Science Inc.) and complement C3 component (AssayPro) were performed following manufacter’s instructions. Assays were performed by duplicate in each sample used in 2D-DIGE proteomic analysis. Results were normalized by the total protein concentration. Data are presented as μg (studied protein)/μg (total protein).

### Milliplex MAP assays

Two MILLIPLEX^TM^ MAP assays (Millipore) were done. Apolipoproteins apoAI, apoCIII, apoE and the acute-phase protein serum amyloid A (SAA) were measured. Analyses were performed as manufacturer suggested and HDL dilutions were done in function of the sensibility of the panel. Assays were performed by duplicate in each sample used in 2D-DIGE proteomic analysis. Results were normalized by the total protein concentration. Data are presented as μg (studied protein)/μg (total protein).

### Statistical analysis

Continuous clinic and biochemical variables and protein levels of ELISA and MILLIPLEX MAP assays were expressed as median (interquartile range). Differences in median values were assessed using Wilcoxon test. All statistical analyses were performed with SPSS software (version 15.0), with significance set at p < 0.05.

## Competing interests

None of the authors declared a conflict of interest.

## Authors’ contributions

EB carried out the proteomic studies, the immunoassays and drafted the manuscript. RMG carried out the subject’s selection and participated in the collection of data. AC participated in the design of the study, performed the statistical analysis and corrected the draft. SF participated in the inmunoassays analysis and corrected the English. AB collected the data and helped into the subject’s selection and administrated the omega-3 supplementation. IJ and JV carried out the mass spectrometry analysis. JV helped in the coordination of the proteomic analysis and in the draft corrections. FC conceived of the study, and participated in its design and coordination and helped to draft the manuscript. All authors read and approved the final manuscript.

## Supplementary Material

Additional file 1 **Normalized volumes of differentially expressed protein spot-features.** The file is a table that shows the normalized volumes of the spots that resulted statistically different in their expression. (DOC 72 kb) Click here for file
