# Supplementary material for: Beneficial effects of omega-3 fatty acids in the proteome of high-density lipoprotein proteome
Source: Lipids Health Dis. 2012 Sep 16;11:116. doi: 10.1186/1476-511X-11-116 (PMC3480889; doi:10.1186/1476-511X-11-116)
Supplement: Additional file 1 — Normalized volumes of differentially expressed protein spot-features. The file is a table that shows the normalized volumes of the spots that resulted statistically different in their expression. (DOC 72 kb) [file 1476-511X-11-116-S1.doc]

Additional File 1: Normalized volumes of differentially expressed protein spot-features

|  |  |  |  |  |  | Normalized spot volume | | | | | | | | | | | |
| --- | --- | --- | --- | --- | --- | --- | --- | --- | --- | --- | --- | --- | --- | --- | --- | --- | --- |
|  |  |  |  |  |  | Basal situation | | | | | | Post ω-3 PUFAs supplementation | | | | | |
| # Spot | Acess number | Identification | *p*Value | Fold Change | Highest media | Gel 1 | Gel 2 | Gel 3 | Gel 4 | Gel 5 | Gel 6 | Gel 1 | Gel 2 | Gel 3 | Gel 4 | Gel 5 | Gel 6 |
| 837 | P02647 | ApoA-I | 0.044 | 1.18 | Post | 1.104 | 0.937 | 1.212 | 1.169 | 1.077 | 0.782 | 1.109 | 1.252 | 1.174 | 1.640 | 1.341 | 0.925 |
| 997 | P10909 | Clusterin | 0.029 | 1.30 | Post | 0.582 | 0.949 | 0.765 | 1.296 | 1.047 | 0.681 | 0.873 | 1.553 | 1.103 | 1.113 | 1.355 | 0.929 |
| 610 | P02675 | Fibrinogen β | 0.048 | 1.10 | Post | 0.833 | 0.894 | 0.826 | 0.483 | 1.308 | 1.298 | 1.052 | 0.963 | 0.991 | 0.613 | 1.326 | 1.273 |
| 612 | P02675 | Fibrinogen β | 0.045 | 1.10 | Post | 1.021 | 0.970 | 0.775 | 0.475 | 1.360 | 1.328 | 1.307 | 0.968 | 0.930 | 0.539 | 1.372 | 1.435 |
| 721 | P10909 | Clusterin | 0.020 | 1.40 | Post | 0.457 | 0.721 | 2.239 | 0.958 | 0.804 | 0.675 | 0.729 | 1.227 | 3.321 | 0.863 | 0.996 | 1.037 |
|  | P02647 | ApoA-I |  |  |  |  |  |  |  |  |  |  |  |  |  |  |  |
|  | P00739 | Haptoglobin-related protein |  |  |  |  |  |  |  |  |  |  |  |  |  |  |  |
| 732 | P00739 | Haptoglobin-related protein | 0.015 | 1.37 | Post | 0.378 | 0.775 | 2.840 | 0.656 | 0.634 | 0.379 | 0.591 | 1.011 | 4.114 | 0.636 | 0.756 | 0.625 |
| 965 | P27169 | Serum paraoxonase | 0.003 | 1.60 | Post | 0.943 | 0.963 | 0.701 | 0.673 | 0.768 | 0.907 | 1.593 | 1.397 | 1.510 | 0.812 | 1.327 | 1.276 |
| 970 | P27169 | Serum paraoxonase | 0.000 | 1.97 | Post | 0.786 | 0.735 | 0.674 | 0.454 | 0.723 | 0.703 | 1.534 | 1.489 | 1.584 | 0.716 | 1.534 | 1.153 |
| 973 | P27169 | Serum paraoxonase | 0.000 | 1.86 | Post | 0.721 | 0.794 | 0.712 | 0.490 | 0.831 | 0.736 | 1.420 | 1.517 | 1.552 | 0.707 | 1.632 | 1.143 |
| 825 | P01834 | Ig kappa chain C region | 0.036 | 1.27 | Post | 2.148 | 0.644 | 2.091 | 1.156 | 1.673 | 1.282 | 2.561 | 0.732 | 3.266 | 1.233 | 1.709 | 1.915 |
|  | P02647 | Apolipoprotein A-I |  |  |  |  |  |  |  |  |  |  |  |  |  |  |  |
| 578 | P01009 | Alpha-1-antitrypsin | 0.001 | 1.35 | Basal | 2.254 | 1.574 | 0.986 | 1.295 | 1.039 | 0.972 | 1.546 | 1.153 | 0.881 | 0.902 | 0.733 | 0.779 |
| 986 | P00736 | Complement C1r subcomponent | 0.018 | 1.18 | Basal | 0.952 | 1.353 | 1.070 | 0.979 | 1.206 | 1.110 | 0.926 | 1.139 | 0.841 | 0.771 | 1.167 | 0.789 |
| 987 | P02768 | Serum Albumin | 0.032 | 1.17 | Basal | 1.142 | 1.647 | 0.881 | 0.807 | 1.218 | 0.952 | 1.180 | 1.251 | 0.772 | 0.722 | 1.077 | 0.679 |
|  | P01871 | Ig mu chain C region |  |  |  |  |  |  |  |  |  |  |  |  |  |  |  |
|  | P00736 | Complement C1r subcomponent |  |  |  |  |  |  |  |  |  |  |  |  |  |  |  |
| 324 | P08603 | Complement factor H | 0.017 | 1.39 | Basal | 1.525 | 1.206 | 1.129 | 1.007 | 1.011 | 0.923 | 0.888 | 0.650 | 0.804 | 0.888 | 0.847 | 0.834 |
| 980 | P02751 | Fibronectin | 0.010 | 1.50 | Basal | 1.058 | 1.099 | 1.116 | 1.652 | 1.822 | 0.992 | 1.021 | 0.706 | 0.865 | 0.926 | 0.918 | 0.711 |
| 981 | P02751 | Fibronectin | 0.002 | 1.48 | Basal | 1.263 | 1.571 | 1.099 | 1.421 | 1.733 | 0.938 | 1.070 | 0.980 | 0.816 | 0.924 | 0.954 | 0.674 |

The normalized volume of the differentially expressed and identified spots in each 2D-DIGE gel is shown in the Table.
